# Pramipexole Impairs Stimulus-Response Learning in Healthy Young Adults

**DOI:** 10.3389/fnins.2016.00374

**Published:** 2016-08-19

**Authors:** Haley Gallant, Andrew Vo, Ken N. Seergobin, Penny A. MacDonald

**Affiliations:** ^1^The Brain and Mind Institute, University of Western OntarioLondon, ON, Canada; ^2^Department of Psychology, University of Western OntarioLondon, ON, Canada; ^3^Department of Clinical Neurological Sciences, University of Western OntarioLondon, ON, Canada

**Keywords:** stimulus-response learning, dopamine agonist, pramipexole, Parkinson's disease, dopamine overdose hypothesis

## Abstract

Dopaminergic therapy has paradoxical effects on cognition in Parkinson's disease (PD) patients, with some functions worsened and others improved. The dopamine overdose hypothesis is proposed as an explanation for these opposing effects of medication taking into account the varying levels of dopamine within different brain regions in PD. The detrimental effects of medication on cognition have been attributed to exogenous dopamine overdose in brain regions with spared dopamine levels in PD. It has been demonstrated that learning is most commonly worsened by dopaminergic medication. The current study aimed to investigate whether the medication-related learning impairment exhibited in PD patients is due to a main effect of medication by evaluating the dopamine overdose hypothesis in healthy young adults. Using a randomized, double-blind, placebo-controlled design, 40 healthy young undergraduate students completed a stimulus-response learning task. Half of the participants were treated with 0.5 mg of pramipexole, a dopamine agonist, whereas the other half were treated with a placebo. We found that stimulus-response learning was significantly impaired in participants on pramipexole relative to placebo controls. These findings are consistent with the dopamine overdose hypothesis and suggest that dopaminergic medication impairs learning independent of PD pathology. Our results have important clinical implications for conditions treated with pramipexole, particularly PD, restless leg syndrome, some forms of dystonia, and potentially depression.

## Introduction

Parkinson's disease (PD) is a progressive neurodegenerative illness, characterized predominantly by motor symptoms including tremor, rigidity, and bradykinesia (Jankovic, 2008). More recently, deficits in cognition have also been described in PD patients (Dubois and Pillon, 1997). Dopaminergic medications, such as levodopa (L-3,4-dihydroxyphenylalanine) or dopamine receptor agonists, are prescribed with the main therapeutic goal of improving motor deficits in PD patients (Hornykiewicz, 1974). However, the effects of dopaminergic therapy on cognition are paradoxical, such that some aspects of cognition are improved whereas others are impaired (Gotham et al., 1988; Cools et al., 2001; MacDonald and Monchi, 2011).

Differences in endogenous dopamine levels within disparate brain regions, related to PD pathophysiology, have been proposed to account for these opposing effects of medication on cognitive functions (Cools, 2006; MacDonald and Monchi, 2011). There is a progressive loss of dopaminergic neurons in the substantia nigra (SN) resulting in dopamine depletion in the dorsal striatum (DS), the brain region that it innervates almost exclusively (Kish et al., 1988). This selective dopamine deficiency in the SN is associated with the emergence of motor impairments in PD but has also been related to deficits in DS-mediated cognitive functions such as selective attention and responding as well as task switching (Cools et al., 2001; MacDonald et al., 2011; MacDonald and Monchi, 2011). In contrast, dopaminergic neurons within the ventral tegmental area (VTA) are relatively spared in PD patients (Haber and Fudge, 1997). Subsequently, brain regions innervated by the VTA, such as the ventral striatum, prefrontal and limbic cortices, have relatively spared levels of endogenous dopamine, especially at early disease stages (Haber and Fudge, 1997). Understanding this pathophysiology, it has been contended that cognitive operations mediated by VTA-innervated brain regions, such as probabilistic reversal learning, learning associations between stimuli, as well as stimuli and responses, remain unaffected at baseline due to relatively replete dopamine levels (Cools et al., 2001; Cools, 2006; MacDonald et al., 2011; MacDonald and Monchi, 2011). The differential levels of endogenous dopamine at baseline within the DS compared to the VTA-innervated brain regions have provided a foundation to explain the paradoxical effects of dopaminergic medication on cognition (Cools, 2006; MacDonald and Monchi, 2011).

This dopamine overdose hypothesis has been proposed as an explanation for the differential effects of dopaminergic therapy on disparate cognitive functions (Gotham et al., 1988; Cools, 2006; MacDonald and Monchi, 2011). Dopaminergic medications are titrated to motor symptoms, which are improved by restoring the maximally dopamine-depleted DS (Hornykiewicz, 1974). These medication doses, however, overdose VTA-innervated brain regions, disrupting their functions (Gotham et al., 1988; Swainson et al., 2000; Cools et al., 2001, 2007; MacDonald et al., 2013b; Hiebert et al., 2014b). Accordingly, DS-mediated cognitive functions are improved whereas dopaminergic therapy has detrimental effects on functions mediated by relatively replete VTA-innervated brain regions (Cools et al., 2001; MacDonald and Monchi, 2011). In support of this, many studies have consistently shown that dopaminergic medication improves PD patient performance on various DS-mediated tasks (Cools et al., 2001, 2003; MacDonald et al., 2011; Aarts et al., 2014) but impairs performance on tasks associated with VTA-innervated brain regions (Swainson et al., 2000; Cools et al., 2001; MacDonald et al., 2011).

Learning is the cognitive operation that appears to be most commonly worsened by dopaminergic therapy. Exploring performance on various learning tasks in PD patients on relative to off medication has demonstrated therapy-related impairments in stimulus-reward reversal (Swainson et al., 2000; MacDonald et al., 2013b), sequence (Feigin et al., 2003), probabilistic classification (Jahanshahi et al., 2010), abstract figure, and list learning (MacDonald et al., 2013a). Further, stimulus-response learning is impaired in PD patients on compared to off dopaminergic therapy, but patients off medication exhibit comparable learning to healthy age-matched controls (Vo et al., 2014). In support of this, Hiebert et al. (2014a) used functional magnetic resonance imaging (fMRI) to investigate the brain regions involved in stimulus-response learning in healthy young adults. They reported that learning stimulus-response associations through feedback is mediated by the ventral striatum (Hiebert et al., 2014a), a VTA-innervated brain region (Haber and Fudge, 1997), explaining the effects of dopaminergic therapy on stimulus-response learning in PD and supporting the dopamine overdose hypothesis (Vo et al., 2014).

Overwhelmingly, investigations of the dopamine overdose hypothesis are performed in PD patients. This strategy, however, cannot resolve whether this is a main effect of dopaminergic medications or whether dopamine overdose is the result of a dopaminergic medication by PD pathology interaction. We hypothesized that dopaminergic medication impairs learning independent of PD pathology. A critical test of the dopamine overdose hypothesis consequently involves testing the effect of dopaminergic therapy on functions in healthy individuals (Vo et al., 2016). Healthy young adults provide an ideal model to investigate whether learning impairment is simply due to dopamine overdose in VTA-innervated brain regions because they have optimal endogenous dopamine levels presumably (Haber and Fudge, 1997). The use of healthy young participants eliminates a number of confounds that exist in PD such as dopamine receptor sensitization secondary to chronic exposure (Voon et al., 2009) as well as altered synaptic regulation of dopamine in the form of decreased dopamine transporter (DAT), which also occurs in PD (Harrington et al., 1996; Kordower et al., 2013). In brief, if the mechanism for the previously reported deficits in stimulus-response learning in PD (Vo et al., 2014) is the straightforward result of dopamine overdose to VTA-innervated brain regions (Hiebert et al., 2014a), we expect dopaminergic therapy-related impairments in healthy young adults that parallel effects in PD. In this respect, studying medication effects on learning in healthy individuals provides a critical test of the prevalent dopamine overdose hypothesis.

Using a modified version of the stimulus-response learning task, which previously demonstrated impairment in PD patients on dopaminergic medication (Vo et al., 2014), we compared the effects of pramipexole, a dopamine agonist, versus placebo on stimulus-response learning in healthy young adults. We predicted that participants treated with pramipexole would exhibit impaired learning relative to placebo controls.

## Materials and methods

### Participants

Forty-five healthy young adults (29 females, 16 males) ranging in age from 18 to 23 years (*M* = 20.69 years, *SEM* = 0.17) participated in the present experiment. All individuals reported no history of previous psychiatric or neurological disorders. Individuals with current or past alcohol, prescription, or recreational drug abuse, or those who were taking cognitive-enhancing medications (e.g., Methylphenidate, Donepezil, Rivastigmine, Galantamine, or Memantine) were excluded from participating. No participants presented with contraindications for pramipexole (e.g., hypotension or treatment with neuroleptics). No participant had prior experience taking pramipexole. One individual met the exclusion criteria of previous drug use and 4 participants withdrew from the study due to adverse side effects (e.g., nausea, vomiting). Thus, 40 individuals (24 females, 16 males) were included in the final data analysis.

This study was approved by the Health Sciences Research Ethics Board of the University of Western Ontario. Prior to testing, all participants provided informed written consent to the approved protocol according to the Declaration of Helsinki (World Medical Association, 2013). Participants were compensated for their participation.

### Affective and cognitive measures

Prior to the experimental task, participants completed a series of written standardized cognitive and affective measures to screen for pre-existing differences between our groups. Anxiety, depression, and apathy were measured using the Beck Anxiety Inventory (BAI, Beck and Steer, 1990), Beck Depression Inventory-II (BDI-II, Beck et al., 1996), and Starkstein Apathy Scale (Starkstein et al., 1992), respectively. The Montreal Cognitive Assessment (MOCA, Nasreddine et al., 2005), which includes measures of memory, executive functioning, attention, visuospatial ability, abstraction, language, concentration, calculation, and orientation, was used to screen for cognitive impairment through both written and verbal tasks. Verbal fluency was assessed by the total number of words beginning with the letter F provided by the participant in 1 min as part of the MOCA (Nelson and Willison, 1991). Visuo-constructional skills were compared using scores on the 3D cube and clock drawing subsections of the MOCA (Nelson and Willison, 1991). Finally, verbal IQ scores were computed using the National Adult Reading Test (NART, Nelson and Willison, 1991), which is a standardized measure of adult intelligence.

### Physiological and mood rating measures

Measurements of heart rate, blood pressure, and alertness (Bond and Lader, 1974) were collected three times during the experiment: prior to capsule administration (i.e., pre-administration), following a wait period but before beginning the task (i.e., pre-test), and after completion of the task (i.e., post-test). This allowed for assessment of the possible physiological effects and changes to subjective mood attributable to pramipexole.

### Apparatus

The experiment was conducted on the Windows 7 operating system. The computer monitor was placed ~50 cm away from the participant for optimal viewing. A standard keyboard was used to record key-press responses during the task.

### Stimuli

**Figure 1:**
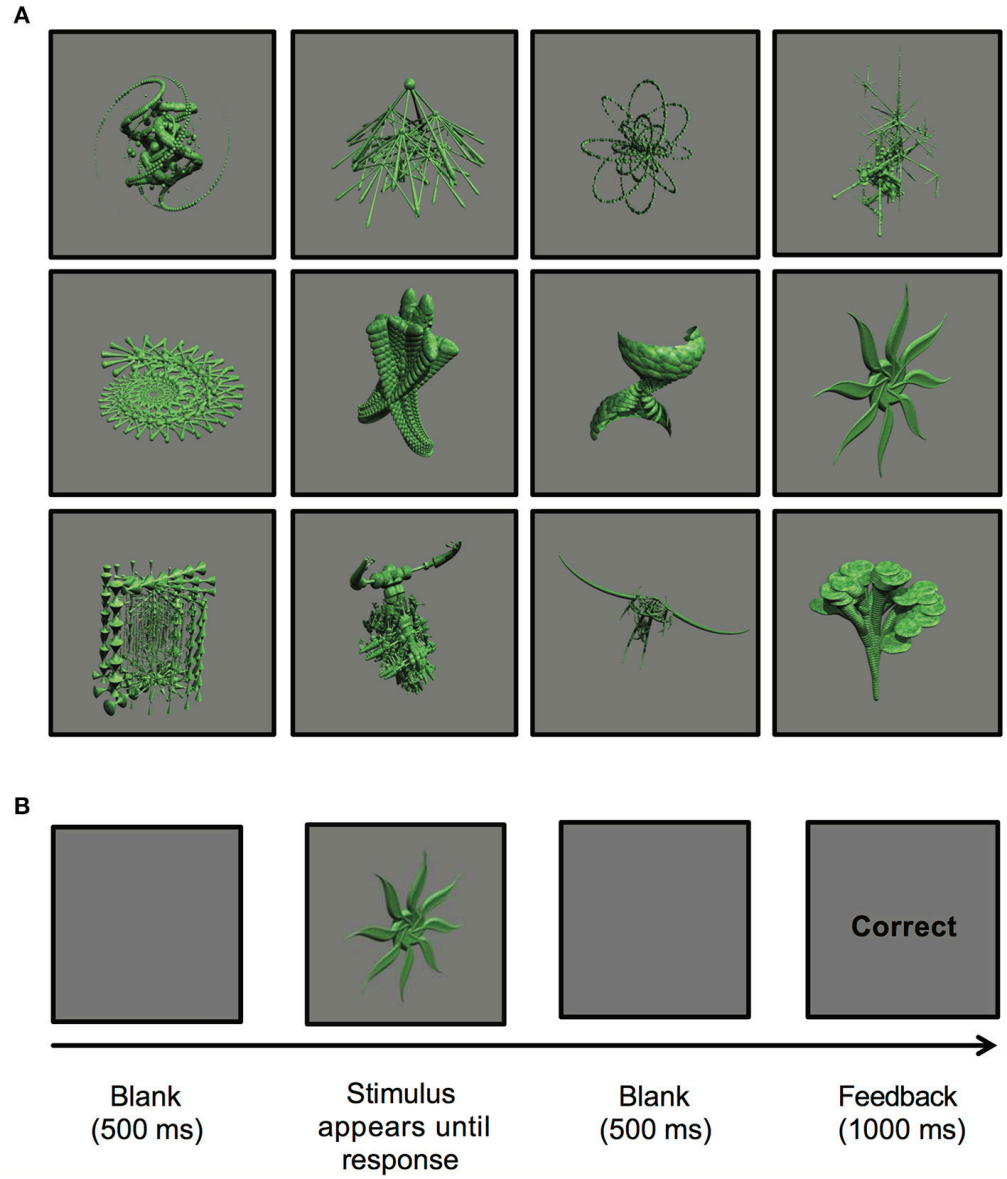
**(A)** Computer-generated abstract images presented during the stimulus-response learning task (*GroBoto*, Braid Art Labs, Colorado Springs, USA). **(B)** An example of a single stimulus-response learning trial.

The stimuli presented in the experiment were comprised of 12 abstract images. These images were computer-generated using *GroBoto* (Braid Art Labs, Colorado Springs, USA) and are displayed in Figure 1A.

### Design and procedure

All participants completed a testing session lasting ~3 h. To prevent interference with drug absorption, participants refrained from caffeine, alcohol, and protein-rich foods on the day of testing and from eating within 1 h before the start of testing. Participants were randomly assigned to either the drug or placebo condition, with half of participants receiving 0.5 mg of pramipexole and the other half receiving an equal volume of placebo. The dose used was selected based on previous psychopharmacological studies of pramipexole in healthy adults (Pizzagalli et al., 2008; Santesso et al., 2009). Both drug and placebo were administered orally in identical capsules to achieve double-blindness. Capsule preparation and randomization were performed by a third party who was not implicated in testing participants. Cognitive testing commenced ~2 h following ingestion of either capsule to ensure that peak plasma pramipexole concentrations were reached (Wright et al., 1997). Prior to beginning the experimental task, participants completed a series of practice trials to ensure that they understood the task instructions.

During the experimental task, participants learned to associate 12 abstract images with one of three possible key-press responses. Four images were assigned to each key (labeled “1,” “2,” or “3”). On each trial, an image was presented and remained in the center of the computer screen until the participant performed a key-press response of either “1,” “2,” or “3” with their index, middle, or ring finger of their dominant hand. Following each key-press response, outcome feedback of either “Correct” or “Incorrect” was provided. This feedback allowed for individuals to learn associations among images and key-press responses through trial and error. Participants were informed that the associations between images and key-press responses did not change between trials, thus allowing for learning to occur.

Each trial proceeded as follows: (a) a single image was displayed until a key-press response was made; (b) a blank screen was presented for 500 ms; (c) either “Correct” or “Incorrect” outcome feedback was displayed for 1000 ms; (d) a blank screen appeared for 500 ms to separate trials (see Figure 1B).

The experimental task was divided into five blocks of 24 trials each. Each of the 12 abstract images was presented twice per block in random order. At the end of each block, participants were provided a percent accuracy score to summarize their learning performance.

### Data analysis

For all demographic, cognitive, and affective control measures, data were analyzed using independent *t*-tests contrasting scores to compare pramipexole and placebo groups. For physiological and alertness measures, we performed separate 2 × 3 mixed ANOVAs with Group (Pramipexole vs. Placebo) as the between-subject factor and Time (Pre-administration vs. Pre-test vs. Post-test) as the within-subject variable on heart rate, systolic blood pressure, diastolic blood pressure, and alertness scores. To assess stimulus-response learning, we performed separate 2 × 5 mixed ANOVAs with Group (Pramipexole vs. Placebo) as the between-subject factor and Block (1 vs. 2 vs. 3 vs. 4 vs. 5) as the within-subject variable on accuracy and response time (RT) scores. Higher accuracy scores and faster RTs on each block indicated more efficient learning.

## Results

### Demographic, affective, and cognitive control measures

Group demographics, including age and education level, as well as scores on cognitive and affective measures, are reported in Table 1. Independent *t*-tests revealed no significant demographic, affective, or cognitive differences between pramipexole and placebo groups (all *p* > 0.05; see Table 1).

**Table 1:** **Demographic, cognitive, and affective measures for pramipexole and placebo groups**.

**Measure**	**Placebo (*n* = 20; 12 females)**	**Pramipexole (*n* = 20; 12 females)**	***p*-value**
Age	20.50 (0.29)	20.80 (0.21)	0.405
Education	15.40 (0.23)	15.55 (0.20)	0.628
NART	118.95 (1.45)	118.71 (0.99)	0.892
MOCA	27.80 (0.34)	27.80 (0.43)	1.000
F-words	14.00 (1.02)	12.75 (0.83)	0.347
Clock	2.95 (0.05)	2.90 (0.07)	0.560
Cube	0.85 (0.08)	0.90 (0.69)	0.643
BDI-II	9.60 (1.61)	8.05 (1.18)	0.441
BAI	8.60 (1.85)	7.45 (1.38)	0.622
Apathy	11.50 (1.07)	11.20 (0.77)	0.821

*Mean values (±SEM) are reported. Education is measured in years. NART, National Adult Reading Test indicates IQ score. The Montreal Cognitive Assessment (MOCA) is scored out of 30, Clock is scored out of 3 and Cube is scored out of 1. BDI-II, Beck Depression Inventory II is scored out of 63; BAI, Beck Anxiety Inventory is scored out of 63. Apathy is scored out of 42*.

### Physiological measures, mood ratings, and predictions

**Figure 2:**
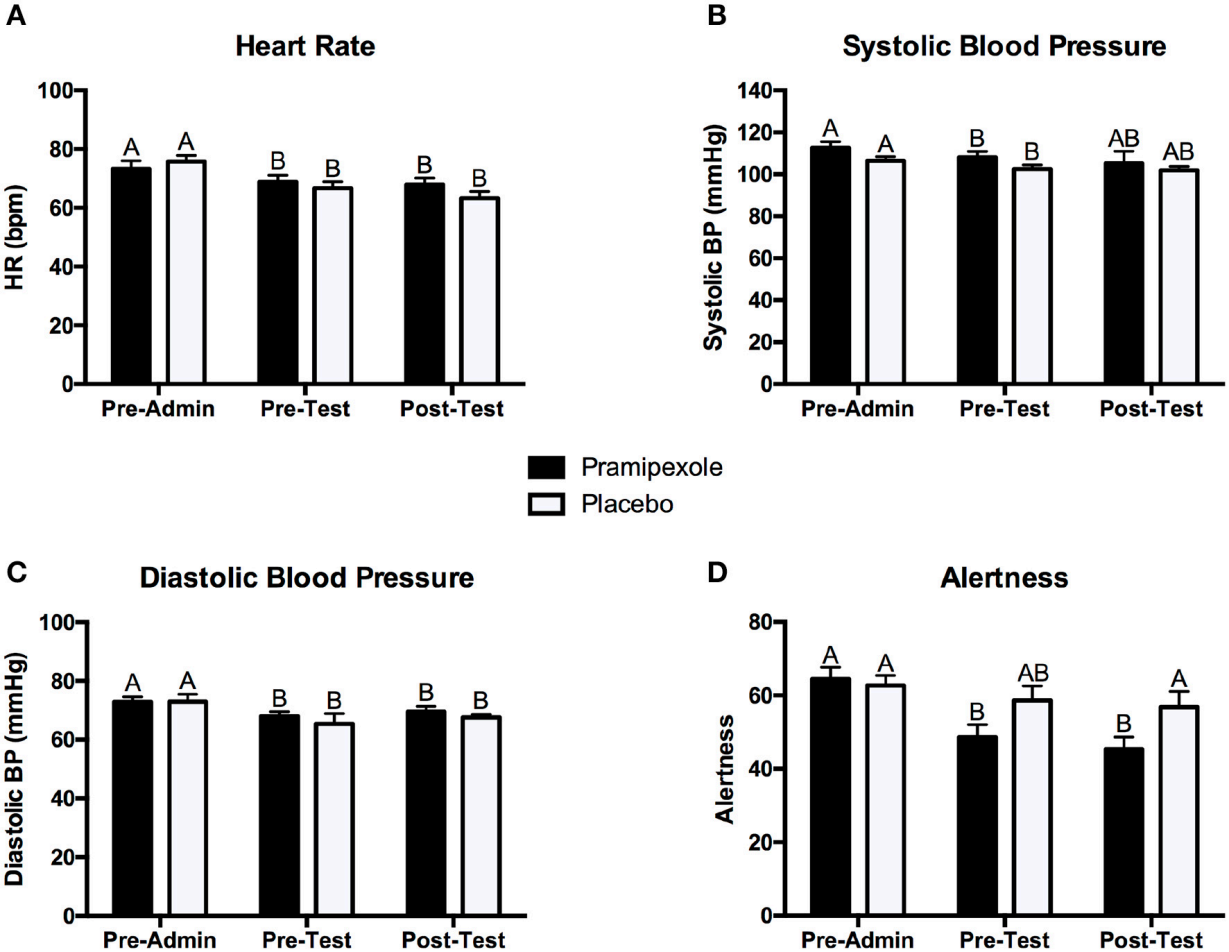
**Mean (A) heart rate, (B) systolic blood pressure, (C) diastolic blood pressure, and (D) alertness scores for the pramipexole and placebo groups at pre-administration, pre-test, and post-test**. Error bars represent standard error of the mean. Means with different letters are significantly different; *p* < 0.05.

To assess the physiological and subjective mood effects of pramipexole, we conducted separate 2 × 3 mixed ANOVAs on heart rate, systolic blood pressure, diastolic blood pressure, and alertness. Figure 2 displays the mean heart rate (see Figure 2A), systolic blood pressure (see Figure 2B), diastolic blood pressure (see Figure 2C), and alertness scores (Figure 2D) measured pre-administration, pre-test, and post-test for the pramipexole and placebo groups.

In regards to heart rate, we found a significant main effect of Time [*F*_(2, 74)_ = 32.40, *MSE* = 25.12, *p* < 0.001]. *Post-hoc* pairwise comparisons revealed that heart rate pre-administration was significantly higher than at both pre-test and post-test (both *p* < 0.001; see Figure 2A). However, there was no significant difference in heart rate between the pre-test and post-test period (*p* = 0.281). There was no significant main effect of Group on heart rate [*F*_(1, 37)_ = 0.084, *MSE* = 269.69, *p* = 0.774]. We did find a significant Group × Time interaction effect [*F*_(2, 74)_ = 4.369, *MSE* = 25.12, *p* = 0.016]. *Post-hoc* pairwise comparisons demonstrated that heart rate was significant lower at pre-test (*p* = 0.035) and post-test (*p* = 0.010) compared to pre-administration (see Figure 2A) for the pramipexole group, but that pre-test and post-test measures did not significantly differ (*p* = 1.000). Similarly, heart rate was significantly higher at pre-test (*p* < 0.001) and post-test (*p* < 0.001) compared to pre-administration for the placebo group, but pre-test and post-test measures did not significantly differ (*p* = 0.275). These findings indicate that the interaction effect was driven by Time and not by Group.

Analysis of systolic blood pressure revealed a significant main effect of Time [*F*_(2, 74)_ = 3.541, *MSE* = 109.97, *p* = 0.034; see Figure 2B]. *Post-hoc* tests demonstrated that systolic blood pressure pre-administration was significantly higher than at pre-test measure (*p* = 0.005) and there was no significant difference between pre-administration and post-test (*p* = 0.090) or pre-test and post-test measurements (*p* = 1.000). There was neither a significant main effect of Group [*F*_(1, 37)_ = 1.821, *MSE* = 392.94, *p* = 0.185] nor a significant Group × Time interaction effect [*F*_(2, 74)_ = 0.192, *MSE* = 109.97, *p* = 0.825].

In regards to diastolic blood pressure, we found a significant main effect of Time [*F*_(2, 74)_ = 5.167, *MSE* = 59.78, *p* = 0.008; see Figure 2C]. *Post-hoc* tests revealed that pre-administration diastolic blood pressure was significantly higher than at both pre-test (*p* = 0.035) and post-test (*p* = 0.034), and that pre-test and post-test systolic blood pressure did not differ significantly (*p* = 0.784). Lastly, we found neither a significant main effect of Group [*F*_(1, 37)_ = 0.739, *MSE* = 135.98, *p* = 0.395] nor a significant Group × Time interaction effect [*F*_(2, 74)_ = 0.073, *MSE* = 59.78, *p* = 0.930].

For alertness scores, we found a significant main effect of Time [*F*_(2, 76)_ = 13.650, *MSE* = 128.09, *p* < 0.001]. Pairwise comparisons revealed that pre-administration alertness scores were significantly higher compared to both pre-test (*p* = 0.001) and post-test (*p* < 0.001), but there was no significant difference in pre-test and post-test scores (*p* = 0.926). There was no significant main effect of Group [*F*_(1, 38)_ = 2.628, *MSE* = 496.90, *p* = 0.113]. We found a significant Group × Time interaction effect [*F*_(2, 76)_ = 4.111, *MSE* = 128.09, *p* = 0.020], however, reflecting greater change in alertness for pre- and post-test relative to pre-administration scores for the pramipexole group compared to the placebo group. Examining simple effects, these differences relative to pre-administration reached significance for the pramipexole group (*p* < 0.001 for both). Though the effect of Time on alertness was large, η^2^ = 0.264, the Group x Time effect was relatively small, η^2^ = 0.098. We attribute the main effect of Time on alertness, collapsed across Group, to increased comfort with the testing situation as well as some fatigue associated with completing a number of demographic, clinical, and affective questionnaires. The smaller effect of Group × Time, however, reflected somewhat sedating property of pramipexole (Micallef-Roll et al., 2001; Micallef et al., 2009). Despite these differences across Time and related to pramipexole treatment, at no point in the experiment did either group rate their level of alertness as significantly somnolent (lowest mean alertness score was 45.31 on a 100-point scale). Potentially owing to the somewhat sedating effect of pramipexole, despite double blinding, at the end of the experiment, participants' deduced their assigned group (i.e., pramipexole vs. placebo) with an accuracy of 72.5%.

### Learning of stimulus-response associations

**Figure 3:**
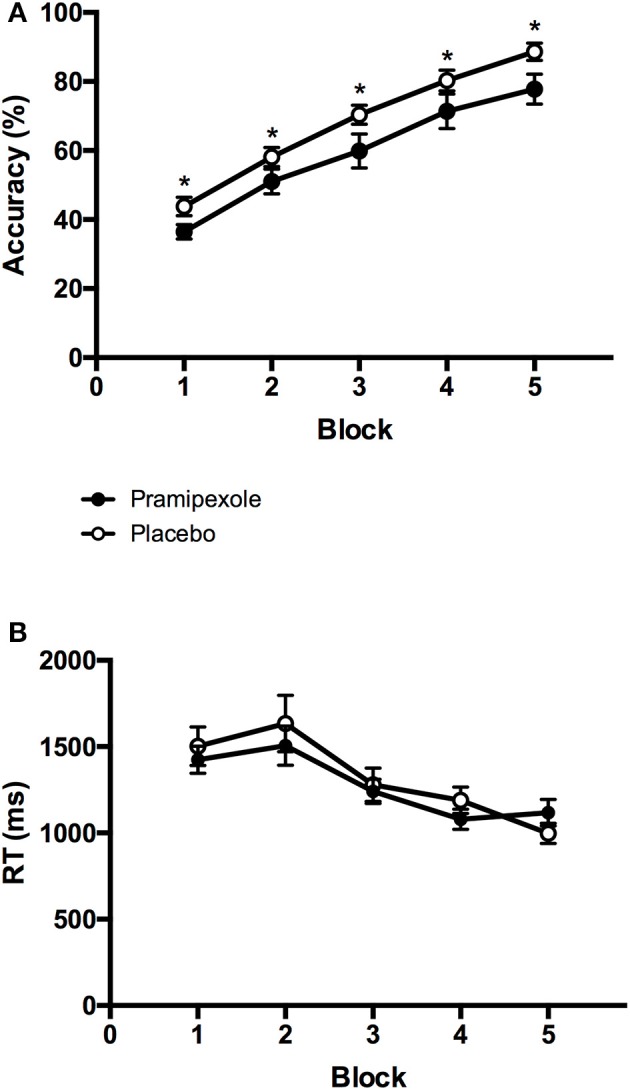
**Mean (A) percent accuracy scores and (B) key-press response times from each block for the pramipexole and placebo groups**. Error bars represent standard error of the mean. Significant differences between the two groups are marked with asterisks (*p* < 0.05).

To compare learning performance between experimental groups and across blocks, we performed a 2 × 5 mixed ANOVA on accuracy scores. Figure 3A displays the mean accuracy scores on each block (1-5) for the pramipexole and placebo groups separately. We found a significant main effect of Block [*F*_(4, 152)_ = 91.648, *MSE* = 0.01, *p* < 0.001; η^2^ = 0.707]. *Post-hoc* pairwise comparisons revealed significant differences in accuracy across all five blocks such that accuracy scores improved with each subsequent block (all *p* < 0.001) as expected. There was also a significant main effect of Group [*F*_(1, 38)_ = 5.430, *MSE* = 0.07, *p* = 0.025; see Figure 3A], with significantly lower accuracy scores in the pramipexole compared to the placebo group across blocks. The effect size was moderate to large (η^2^ = 0.125). Finally, there was no significant Group × Block interaction effect [*F*_(4, 152)_ = 0.238, *MSE* = 0.01, *p* = 0.916].

To further investigate the possibility that differences in learning performance between pramipexole and placebo groups were simply due to medication-induced decreases in alertness, we conducted an additional 2 × 5 mixed ANOVA on key-press RTs. Mean RTs for each block (1–5) are plotted for the pramipexole and placebo groups separately in Figure 3B. There was a significant main effect of Block [*F*_(4, 152)_ = 24.593, *MSE* = 76416.75, *p* < 0.001]. *Post-hoc* tests revealed that RTs during Blocks 4 and 5 were significantly faster compared to the first three blocks, and Block 3 was significantly faster than the initial two blocks (both *p* < 0.05). There were no statistically significant differences between Blocks 1 and 2 (*p* = 1.000) or between Blocks 4 and 5 (*p* = 0.196), however. Most important though, we found no significant main effect of Group [*F*_(1, 38)_ = 0.181, *MSE* = 609848.42, *p* = 0.673], as pramipexole and placebo control groups did not differ in their RTs. There was also no significant Group × Block interaction effect [*F*_(4, 152)_ = 1.293, *MSE* = 76416.75, *p* = 0.275].

## Discussion

In the present study, we demonstrated that a single dose of pramipexole, a dopamine agonist, impairs stimulus-response learning in healthy young adults. This was indicated by lower overall accuracy scores on a stimulus-response learning task in the pramipexole group relative to a matched placebo control group. We found that across blocks of this task, mean percent accuracy scores increased, and mean key-press RTs decreased for both groups, thus demonstrating that learning was achieved. The differential learning performance between the pramipexole and placebo groups was not due to pre-existing cognitive, affective, or demographic differences as no significant differences in any of these control measures were found. In addition, there were no significant differences in physiological measures of heart rate, systolic blood pressure or diastolic blood pressure between the two groups at any time point. Thus, the learning impairment by medication was not a consequence of the physiological effects of the drug. Although heart rate and blood pressure significantly decreased throughout the experiment, this occurred in both groups and we attributed this finding to increasing comfort with the testing situation and perhaps an element of general fatigue and prolonged sitting over the experimental session. This was not due to pramipexole, however, as there were no group differences in these measures.

Alertness was reduced over time, collapsed across group, from the pre-administration to the pre-test and post-test periods. Although there was not a main effect of Group on alertness scores, there was a Group × Time interaction, with lower alertness scores for participants in the pramipexole group relative to the placebo group at the pre-test and post-test time points. Sedation is a recognized side effect of pramipexole and others have demonstrated clinically significant sedation in young healthy volunteers following administration of dopaminergic medications (Micallef-Roll et al., 2001; Micallef et al., 2009). This potentially accounts for the finding that participants were above chance in accurately judging whether they had received pramipexole or placebo. The effect size for the main effect of Time was large, as suggested by η^2^ = 0.264, which we attribute to increased comfort with the testing situation as well as some fatigue associated with completing a number of demographic, clinical, and affective questionnaires. The effect size for the Group × Time interaction was quite small by comparison (η^2^ = 0.098) and there was no main effect of Group on alertness score. This effect on alertness was smaller than the effect of pramipexole on learning score (η^2^ = 0.125), suggesting that alertness alone may not account for differences in learning between participants receiving pramipexole compared to those receiving placebo. Further, despite these differences across Time and to lesser extent related to pramipexole treatment, at no point in the experiment did either group rate their level of alertness as significantly somnolent (lowest mean alertness score was 45.31 on a 100-point scale). Finally, we found no significant differences in key-press RTs between the two groups during the task. Though there are likely multiple influences on RT, including possibly enhanced stimulus-reward value in the pramipexole group (Hickey et al., 2010), if alertness was significantly compromised due to pramipexole, equivalent RTs across groups would be unexpected. Taken together, we interpret that pramipexole-related learning impairment was likely not entirely attributable to reduced alertness.

The current findings are consistent with the PD literature in demonstrating detrimental effects of dopaminergic medication, including pramipexole, on learning. Many studies have reported various forms of learning impairments in PD patients related to dopaminergic medication, including probabilistic associative (Jahanshahi et al., 2010), list (MacDonald et al., 2013a), and stimulus-reward reversal learning (Swainson et al., 2000; Cools et al., 2001, 2007; MacDonald et al., 2013b). Vo et al. (2014) reported that stimulus-response learning was impaired in PD patients tested on their usual dopaminergic medications (i.e., levocarb and/or dopamine agonists as prescribed by their treating neurologist), compared to when they were tested off medication in a within-subject design. Our results are consistent with this study in demonstrating that pramipexole, a dopamine agonist, impaired learning in a slightly modified version of this same stimulus-response learning paradigm.

As we have done here, investigations of the effects of dopaminergic therapy on cognition have also recently been conducted in healthy controls, to understand the main effect that these medications have on cognition independent of PD pathology (Vo et al., 2016). Our study contributes to this small but growing literature that can determine whether cognitive results are attributable in a straightforward manner to dopaminergic therapy or whether they occur as an interaction between medication and PD pathophysiology. The use of healthy young adults, in particular, provides an ideal control model for exploring the effects of dopaminergic medication on cognition. This strategy is advantageous because it avoids the significant variability in typical PD patient groups related to wide age ranges, large differences in disease severity, medication doses and types (Kalia and Lang, 2015). These studies also can rule out the possibilities that these effects occur only (a) secondary to dopamine receptor sensitization, through chronic exposure to dopaminergic therapy, or (b) due to the fact that PD-associated as well as aging-related reductions in DAT levels, which clears and regulates dopamine at the synapse, predisposing to dopamine overdose (Harrington et al., 1996; Voon et al., 2009; Kordower et al., 2013; Kalia and Lang, 2015). Further, it is important to understand cognitive effects of dopaminergic therapy independent of PD because these medications are used in other conditions such as restless leg syndrome (Liu et al., 2016), in some cases of dystonia (Jankovic, 2013), and are being considered as therapy for depression (Cusin et al., 2013). Finally, if these cognitive effects are main results of dopaminergic therapy, this should alert clinicians to the possibility of cognitive improvements and impairments related to dopaminergic medications at any stage of PD.

Previous studies involving healthy individuals have evaluated the effects of dopamine agonists on learning. These investigations have revealed medication-related impairment in associative (Breitenstein et al., 2006), probabilistic reversal (Mehta et al., 2001), and reinforcement learning (Pizzagalli et al., 2008; Santesso et al., 2009) in healthy adults. Results from the present study are entirely in line with these findings. However, the current results contribute the novel observation that pramipexole impairs stimulus-response learning in healthy young adults, which has not previously been investigated to our knowledge.

The dopamine overdose hypothesis explains the paradoxical effects of dopaminergic therapy on cognition in PD patients as owing to the varying endogenous levels of dopamine in the SN-innervated DS compared to VTA-innervated brain regions. A large literature links learning to VTA-innervated brain regions such as the ventral striatum, hippocampus, and medio-frontal and medio-temporal regions (Schendan et al., 2003; Rudy et al., 2007; Denayer et al., 2008; Talpos et al., 2008; Cavanagh et al., 2010; Guo et al., 2011; Hiebert et al., 2014a; Lungu et al., 2014; Mattfeld and Stark, 2015) and not surprisingly in the PD literature learning is the function vastly, most commonly impaired by dopaminergic therapy. Indeed, with the stimulus-response learning paradigm used here, (a) PD patients were impaired by dopaminergic medication (Vo et al., 2014) and (b) feedback-based learning was shown to be mediated by ventral striatum using fMRI in healthy controls (Hiebert et al., 2014a), strongly supporting this dopamine overdose hypothesis.

In the small literature investigating the effects of dopaminergic therapy in healthy controls, learning is also by far the function most frequently found to be impaired. In healthy young participants, however, all brain regions are dopamine-replete and therefore the straightforward dopamine overdose hypothesis predicts that all cognitive functions that implicate the neurotransmitter dopamine, not just those mediated by VTA-innervated brain regions, should actually worsen with dopaminergic therapy. There should be no specific predilection or vulnerability for learning functions in healthy controls. For example, cognitive functions such as task-set switching (Cools et al., 2001), integration of various influences involved in response selection (MacDonald et al., 2011), set-shifting and conflict monitoring (Monchi et al., 2006; Ko et al., 2009), as well as spatial attention (Christian et al., 2006), Stroop interference (Vernaleken et al., 2007), and spatial working memory (Sawamoto et al., 2008) depend upon dopamine and should all be worsened in the on condition according to the dopamine overdose account. These examples do not exist and indeed there are a very small number of investigations in healthy controls that in fact revealed improvements related to exogenous dopamine administration in short-term spatial memory (Mehta et al., 2001), working memory (Murphy et al., 2016), response inhibition (Roesch-Ely et al., 2005), and novel word learning (Knecht et al., 2004; Shellshear et al., 2015). At this point, it is possible that the effects of dopaminergic therapy on these other cognitive functions have not been investigated. It is equally possible that these investigations have not revealed detrimental effects in the form of between-group or between-condition differences and therefore do not appear in the publication record. Future studies should directly contrast, within the same participants, the effects of dopaminergic therapy on cognitive functions known to be mediated by brain regions innervated by SN and those innervated by VTA, to investigate any particular vulnerabilities. These studies should also include neuroimaging measures to fully substantiate interpretations.

In summary, we have found that a single dose of the dopamine agonist pramipexole impairs stimulus-response learning in healthy young adults who have intact dopamine levels and dopamine regulation functions. This confirms that the detrimental cognitive effects of dopaminergic therapy are a main effect of these drugs and do not arise only as an interaction between these medications and PD pathology. The dopamine overdose hypothesis is supported here. This has implications for use of pramipexole in other conditions such as restless leg, dystonia, or for potential future use of this medication in depression. Further, this alerts clinicians to the potential of detrimental effects of dopaminergic therapy, here pramipexole, on learning in PD patients at all stages of disease, independent of the nature or extent of their PD pathology. The serious motor and cognitive symptoms in PD related to DS dopamine deficiency obviously warrant dopaminergic therapy. However, these results caution that the goal of therapy should be to strike a better balance between these benefits and potential side effects based on individual patient priorities and symptomatology.

## Author contributions

HG participated in data acquisition and interpretation, as well as writing the first draft and revising the manuscript. AV, KS, and PM were involved in study design and conceptualization, obtaining materials, data analysis and interpretation, and revising the manuscript.

## Funding

This research was supported by a Canada Research Chair (CRC) Tier 2 in Cognitive Neuroscience and Neuroimaging to PM (Grant: 950-230372), a Natural Sciences and Engineering Research Council of Canada (NSERC) Discovery Grant to PM (Grant: 6621), an Internal Research Fund Grant from Lawson Research Institute to PM, and an Ontario Graduate Scholarship to AV.

### Conflict of interest statement

The authors declare that the research was conducted in the absence of any commercial or financial relationships that could be construed as a potential conflict of interest.
